# Expert Testimony in Criminal Cases Involving the Plea of Insanity*Read before the Georgia Medical Association, Macon, April, 1897.

**Published:** 1897-06

**Authors:** John C. Olmsted

**Affiliations:** Atlanta, Ga.


ATLANTA

Medical and Surgical Journal.

VOL. XIV. JUNE, 1897. No. 4.

L. B. GRANDY, M.D., MANAGING EDITOR.

M. B. HUTCHINS, M.D., BUSINESS MANAGER.

COLLABORATORS:
A. W. CALHOUN, M.D., LL.D., VIRGIL O. HARDON, M.D., FLOYD W. McRAE, M.D., A. W. STIRLING, M.D., JOSEPH EVE ALLEN, M.D., AUGUSTA, GA., AND HENRY R. SLACK, PH.M., M.D., LAGRANGE, GA.

### ORIGINAL COMMUNICATIONS.

### EXPERT TESTIMONY IN CRIMINAL CASES INVOLVING THE PLEA OF INSANITY.*

BY JOHN C. OLMSTED, M.D.,
ATLANTA, GA.

There are probably no circumstances or conditions under which the medical profession appears to as great disadvantage as those which I have selected as the title of this paper. It is true that much might be said on the same line concerning medico-legal expert testimony in general, especially as we see it illustrated but too often in damage-suit cases, which have almost made the title of doctor, when introduced in such cases, a subject of ridicule and contempt to the legal profession, and of mirth and want of confidence in medical science on the part of the general public. Here, to one who loves his profession, the mortifying spectacle is presented of a certain number of so-called "eminent medical experts" swearing to

*Read before the Georgia Medical Association, Macon, April, 1897.

certain alleged facts, and a number of equally "eminent experts" swearing to the contrary. The resulting impression upon the mind of the court and the enlightenment of the minds of the jury, thus attained, is more easily imagined than described. The demoralizing effect produced, as regards the weight and value to be attached to medical testimony is evident, and it is greatly to be doubted if, as regards the action of the jury, the "expert testimony" exercises much influence, excepting possibly, that of a negative character, in deciding the ultimate result; the appearance of "the doctor" upon the witness stand having been regarded as a pleasant and diverting break in the monotony of the ordinary court proceedings, while it was the signal for the hangers-on and idly curious to press forward to "see the fun."

That this view is not strained or exaggerated, any one familiar with such trials, either as seen in court or as reported by the daily press, must, I think, admit. If such spectacles are thus not infrequently presented by the "medical experts," and, in the character of cases mentioned, are well calculated to bring the science of medicine into derision and contempt, how much more so must this be true in cases of criminal character, where, it may be from the atrocity of the crime committed, the attention of the public is fully aroused and its eye focused upon all of the details of a sensational trial, in which the defense exhausts all the resources of able counsel in endeavoring to establish the existence of insanity, and thus claim that immunity from criminal responsibility which the law affords to those so afflicted, while on the other hand the prosecution seeks to disprove this claim, as the last recourse of ingenious counsel, and insists that the ends of justice, and hence the interests of society, demand a conviction, it may be, of murder. It is, of course, in such cases as this, that the aid of the medical profession is naturally invoked. There can be no disorder of the mind without disorder of the brain. As physiologists and pathologists, we have to study and treat the latter; and for this reason the legislature enacts, that certificates of insanity shall be given by medical men, and by them alone; and that to their care shall be committed those who are insane. Hence it follows that in these cases, involving the gravest questions of criminal responsibility, affecting on the one hand, it may be, the life of a poor, miserable, irresponsible lunatic, and on the other, the interests of society, as influenced, it may be, by the escape through legal subtleties, of an enemy of mankind; it is, I say, in such cases, that to the medical profession the public has a right to look for the conservation of the rights of all concerned, and the vindication of truth and justice. It is here that the preëminence of our noble profession should be most manifest and its benign influence should shine most lustrous. From the calm, dispassionate verdict of medical science, whose "eye single to truth" knows no prejudice, can admit of no sophistry, there should be no appeal.

Is this result obtained, and are the claims of medical science thus sustained, by those who usually appear as its exponents? It is with very great reluctance that I opine that, in the majority of such cases, the answer must be "*No!*" Nor is it difficult to understand how this conclusion is almost insured by the present vicious system of obtaining and presenting medical testimony in such cases. It is not here proposed to enter upon the intrinsic difficulties involved in the medico-legal consideration of the subject of insanity, nor to discuss the legitimate differences of opinion which may occasionally arise between "doctors" in their inductions in a particular case of this most recondite of diseases: these difficulties are well set forth in and known to all familiar with medical jurisprudence. It is rather to another phase of the subject that I would address my remarks, namely, the methods of obtaining and presenting medical testimony, and the attitude usually occupied by the so-called "experts." I use the term "so-called experts" advisedly, for seldom does it happen that the "expert" is really one who can base his claim to a specialistic knowledge upon a broad and deep study of psychological phenomena and familiarity with psychopathological results of medical experience, as formulated in the works of leading alienists. The method of obtaining and presenting "expert testimony" is well known. From the ranks of our profession each side selects certain physicians, either of known preconceived views favorable to their respective interests, or who it is assumed can be induced to entertain and advocate such views. The veteran practitioner and the new-fledged graduate, the profound scholar and man of science, the unprincipled quack and him with the forehead of brass, who holds a ten-dollar "diploma" (perhaps from Wisconsin!), all are presented to the jury with equal dignity and sanction of law, under the comprehensive description of "experts." As has been well said (and that by a lawyer) "under this common guise trickery passes as skill, and assurance as wisdom." "Confidence, in many cases, bears a direct similitude and ratio to their ignorance." Well, however this may be, I contend that the "medical expert," whoever he may be, from the circumstances of the case, and at the very outset of matters, enters into the trial with biased and prejudiced mind, and appears upon the witness-stand as a partizan. That the fair, calm, clear analysis and deliberate conclusion of medical science cannot be obtained under such conditions is, I think, self-evident. Doctors, I assume, like other people, have a good deal of human nature in them, and it must be admitted that under such circumstances the conditions are admirably adapted to induce the "experts" to take a one-sided view of the case. Employed to show one side of a case, it is, perhaps, natural that he should strain every point favorable to his side of a case, and yet look with cold and truly scientific criticism (?) upon everything unfavorable to it. Counter and conflicting testimony among experts, awakens pride of opinion and personal feeling (two elements fatal to the claims of science), the "combat deepens," dogmatic self-assertion, prejudice, ill-formed and hasty conclusions and narrow inductions, take the place of the deliberate, calm, and accurate inductions of science. In fact, it would often seem that the "experts" in their heated zeal, entirely forgot their duty as exponents of science. The matter has with them degenerated into a forensic debate, in which each one is more anxious to achieve a personal victory than concerned for anything else. Considerable "speaking to the galleries" has been observed at times, and evidently appreciated by that not inconsiderable element of the public which regards such professional exhibitions as a "free show." Newspaper interviews, with some "leading experts," are not unknown. On one occasion, in a case of this sort, where quite an array of medical talent had been enlisted, and a good deal of truly scientific opinion elicited, a distinguished "expert" was represented as saying that he

"believed in looking at such cases in a common-sense way." I have yet to learn of any branch of science which ignores the element of "common-sense" in pursuing its particular methods; but it would have been well, in this special application of the term, to have defined what was meant by "common-sense." If the usual meaning was intended, namely, the "common understanding of the mass of the people," I would then remark that if there is any one subject upon which the common people are most notoriously ignorant and ill-informed, it is that of insanity. But to digress no further, as the result of the conflicting testimony of the warring doctors, one thing is apt to be assured, namely, the complete mystification of the jury. The aid which medical science should afford the law, in furthering the ends of justice, is frequently "conspicuous by its absence."

The "medical expert," originally admitted to the courts as *Amicus curiæ* or "friend of the court," to aid the jury in elucidating the truth and arriving at correct conclusions, has now degenerated into the hired advocate in special cases, which he is expected to assist in winning, regardless of all other considerations. Hence, the dignity of the medical profession, and its claims upon the confidence of the public, is greatly weakened.

Having thus, inadequately enough, sketched some of the features of the disease, it may be asked: "What is your remedy?" I am free to say that I have no "cut and dried remedy," preferring to leave the formulation of plans, with their involved details, to the action of this Association. That a remedy is needed, I believe will be conceded, and I would only venture to add, that I think the assembled wisdom of this intelligent body should be directed to the securing of such legislation as would insure the establishment of a medical commission, selected (preferably it seems to me) by the ordinary and grand jury, which commission, by whatever means created, should have the medical decision of all questions involving our profession in such cases. Thus the best medical talent might be obtained, insuring to the law the unprejudiced, calm, and deliberate verdict of medical science. Adequate remuneration should be provided for such service—a matter notoriously needing reform under the present methods, which often compel the attendance of a medical man for days without proper remuneration, and sometimes with none at all.

In conclusion, permit me to say, that if I have borne rather heavily upon my own profession in my remarks, the just occasion for it is sincerely believed to exist, and the need of reform to be manifest. The complaint is made in "the home circle," and I trust that this Association will take such measures to correct the abuses as will advance the interests, influence, dignity and honor of our high calling.

